# Supplementary material for: Use of an inertial sensor and a force platform to assess static balance in participants affected by multibacillary leprosy
Source: PLoS Negl Trop Dis. 2024 Oct 7;18(10):e0012537. doi: 10.1371/journal.pntd.0012537 (PMC11486354; doi:10.1371/journal.pntd.0012537)
Supplement: Attachment — Submitted filename: Response to reviewers v2.docx [file pntd.0012537.s002.docx]

**Methods

Reviewer #1: The purpose of the research was clearly specified.
The population was well selected - although it must be admitted that it is small.
The discussion explains the answer to the research hypothesis.
Statistical calculations correct and nicely described.**

**comments:
The reference group is dominated by men and the study group is dominated by women - does this not affect the results in case of comparisons between groups?**
Authors: Thanks for the comment. Gender-related factors do not interfere with the outcomes, as male and female are affected in the same way by infectious diseases due to physiological issues (ref bellow), such as genetic interactions and sex hormones, which determine whether one sex will be more susceptible to infections, as well as differences in behavioral patterns, indicating greater exposure of either men or women to contagion factors. These facts have been added to the discussion.

Guerra-Silveira F, Abad-Franch F. Sex bias in infectious disease epidemiology: patterns and processes. PLoS One. 2013

**Please add more information about the COM sensor to the article. What was recorded with it, what quantities, how filtered, how processed**
Authors: Thanks for the comment. To begin the test, a sensor was attached to the participant's lower back using a tape, specifically at the 5th lumbar vertebra (L5), as it is close to the body's center of mass. The sensor is a wearable commercial device, containing an embedded triaxial (X, Y and Z axes) accelerometer to measure and record the center of mass (COM) accelerations. This is a 25 mm in diameter x 4 mm ultralight device weighing only 5.6 g, with a replaceable 200 mAH battery with data transfer via a Low-Energy Smart Bluetooth and requires an Android or iOS application (MetaBase, MbientLab, San Francisco, CA, USA) to store the collected data, with a sampling rate set at 100Hz. This information was added to the Methods.

**Reviewer #2:**

**The object of the study is to validate the use of the inertial sensor for leprosy patients through a correlation with the results from a force platform, not the evaluation of the leprosy and healthy subjects as stated by the author at the introduction section.**

Authors: Thanks for the comment. It was adjusted in the new version.

**Also at the introcuction, the author shows references that imply that multibacilary leprosy patients have altered weight bearing disposition due to sensitivity loss, but the sample used in this study had not alteration in sensitivity, since they were all graded 0 (zero) disability, and those with altered sensitivity were graded 1. Therefore, the introduction does not justify the methods used.**

Authors: Thanks for the comment. In the literature, there are descriptions of sensorimotor alterations even in patients without clinical signs or with grade 0 disability, as it is proven that they may also present dysfunctions in isolated nerves and neural impairment (ref bellow). Early identification of neurological alterations in patients with leprosy disease is important for monitoring sensory and motor losses and the consequent disabilities resulting from the disease. This was included in the new introduction.

Nascimento OJM. Leprosy neuropathy: clinical presentations. Arq Neuropsiquiatr 2013;71(9B):661-666

**It was not specifyed if the participants had any level of regular physical activity, since it would impact the results of the inertial sensors and balance control by the hip muscles, and it might explain why the results from MLdisp were not statisticaly different, as opposed to the other variables acquired.**
Authors: Thanks for the comment. During the assessment, participants from both groups reported not engaging in any type of physical activity. This aspect was included in the population characteristics under methods

**--------------------

Results

Reviewer #1: Results presented correctly. unreservedly.
comments:
What does adding graphs over time for one example person contribute to the article - please explain?**

Authors: Thanks for the comment. A representative subject from each group was selected over an estimated period to demonstrate that the AP parameter, compared to ML, exhibits greater oscillations and that under conditions of eyes closed, participants in the Leprosy group show greater oscillations compared to participants in the control group.

**Reviewer #2: In Table 2, it was not specifyed what the value "U/t,df" stands for.**

Authors: Thanks for the comment. It was adjusted in the new version.

**The english grammar should be reviewed for the abstract and the results section.**

Authors: Thanks for the comment. It was adjusted in the new version.

**The sensor validity part of the results section does not describe how the correlation between acceleration (m/s2) and simple range (cm) were made, and does not show data colected from "closed eys" situation. The adition of a table is strongly recommended to elucidade what is mainly visual on this part.**

Authors: Thanks for the comment. The acceleration and simple reach were assessed using the Spearman correlation coefficient for each condition (open eyes and closed eyes). The results from open eyes (OE) and closed eyes (CE) conditions followed a similar pattern (values across the four parameters were correlated in both conditions), hence they were combined in the figure. To address this item a new table (table3) with the specification of both correlations (OE and CE) were included.

**The discussion section re-state that the plantar sensitivity alterations contribute to higher values of COP and COM for the leprosy subject. However, those subjects inluded in this study did not have any sensitivity alteration, which invalidade the choice of sample chosen, and opens up a new question as to why the results were different among the two groups. Is it possible that the degree of disability was not correctly colected?**

Authors: Thanks for the comment. The degree of disability was verified in the evaluation records of leprosy disease patients at the Dermatology Laboratory of the Tropical Medicine Unit, a specialized facility in catering to this population. A second neurological assessment was conducted by the researchers of the present study with these same patients, confirming a disability grade of 0.

**--------------------

Conclusions

Reviewer #1:**

**What COP and COM values are we talking about in the sentence:
"We hypothesized that COP and COM values would be higher in patients with Hansen's disease, and the sensor and platform would indicate group differences in a correlated manner."?**

Authors: Thanks for the comment. The two values found in the four quantitative parameters (TD, AP_disp_, ML_disp_, and AREA) in Table 2, demonstrate in detail that participants with Leprosy present greater oscillations in COP and COM compared to healthy participants. These values were determined by the force platform and correlated with the values found by the sensor in all evaluated parameters, except ML_disp._ We tried to make it clear in the new version.

**Reviewer #2:**

**The limitations of analysis were described as the single acquisition of data with eyes oppend and closed, but I suggest that hte limitation should be that the leprosy sample does not have any sensitivity impairment. It muddles all the means and the discussion of the article**

Authors: Thanks for the comment. Despite the participants exhibiting a disability grade of 0, as previously noted in the literature and described in the introduction, as suggested by the reviewers, they are also susceptible to sensorimotor alterations.

**The conclusions of this study were presented within the Discussion section. It is strongly advised to sepparate them.**

Authors: Thanks for the comment. It was adjusted in the new version.

**--------------------**
