# Supplementary material for: Use of an inertial sensor and a force platform to assess static balance in participants affected by multibacillary leprosy
Source: PLoS Negl Trop Dis. 2024 Oct 7;18(10):e0012537. doi: 10.1371/journal.pntd.0012537 (PMC11486354; doi:10.1371/journal.pntd.0012537)
Supplement: Attachment — Submitted filename: response reviwers _v3.docx [file pntd.0012537.s003.docx]

**Dear authors.
Please make the necessary changes to the text of the manuscript based on the considerations of the reviewers.
Authors can give reasons if they do not agree with the reviewer's suggestion.
I would like to inform you that the title is appropriate.
Best wishes**

Authors: We would like to thank you and the reviewers for the valuable feedback provided on our manuscript. We have carefully reviewed all the comments and have made the necessary corrections where we deemed them appropriate. In particular, we would like to address the third reviewer's suggestion to use a 2x2 ANOVA. After careful consideration, we believe that maintaining our original statistical approach is more suitable for our study. Specifically, the use of non-parametric tests, such as the Mann-Whitney test, was chosen due to the non-normal distribution of our data, which was confirmed by the Shapiro-Wilk test. Repeated measures ANOVA assumes normal distribution and homogeneity of variances, conditions not met by our dataset. Furthermore, our study design involved comparing two independent groups across two conditions, which aligns better with the chosen statistical methods. We hope this explanation clarifies our decision. Both highlighted and clean versions of the revised manuscript are being made available for your review.

**Reviewer #2: The pursue for balance control in leprosy patients might be justified due to the lack of plantar sensitivity. However, the sample of this Work does not have A plantar sensitivity impairment ( being grade 0 for disability measures ). It's My suggestion to the authors to simply verify the balance control of the leprosy patient regardless of the sensitivity, And bring light to other possibilities of balance control for this population, on the discussion section.**

Authors: Thanks for your comment. We have revised the introduction to emphasize the importance of studying balance control in leprosy patients, even in those without apparent plantar sensitivity impairment (grade 0 for disability measures). We have included information about "silent neuropathy" or "quiet nerve paralysis," highlighting that significant sensory and motor impairments can occur in the absence of typical clinical signs such as skin lesions, nerve tenderness, pain, or paraesthesia. This underscores the importance of early identification of l alterations in leprosy patients to monitor and manage sensory and motor deficits, which can prevent the progression to more severe disabilities. We have also expanded the discussion to explore other potential factors that may affect balance control in leprosy patients beyond plantar sensitivity. This includes considering alternative mechanisms such as proprioceptive deficits, changes in neural control, and muscle strength variations that could influence balance in this population. This were included in the new version.

**Reviewer #3: Title is not appropriate. Please edit it**

Authors: We would like to provide our rationale for why we believe the current title, "USE OF AN INERTIAL SENSOR AND A FORCE PLATFORM TO ASSESS STATIC BALANCE IN PARTICIPANTS AFFECTED BY MULTIBACILLARY LEPROSY," is appropriate.

The title accurately reflects the main focus and methodology of our study. It clearly indicates the use of two specific tools—an inertial sensor and a force platform—to evaluate static balance, which is the primary aim of our research. Furthermore, by specifying "participants affected by multibacillary leprosy," the title clearly defines the population under study, ensuring that the scope of the research is immediately apparent to the reader. This title effectively communicates both the methods and the subject of the study, which aligns with the standard practices for titling scientific articles. It ensures that potential readers can easily understand the research focus and target population, which is crucial for the dissemination and impact of our findings. We hope this explanation clarifies the suitability of the chosen title.

**the objectives of the study were clearly articulated with a clear testable hypothesis stated.**

Authors: Thanks.

**Design of this study is case-control not cross-sectional.**

Authors: Thanks for your contribution. We’ve corrected that.

**the population were clearly described and appropriate for the hypothesis being tested.**

Authors: Thanks

**the sample size was sufficient to ensure adequate power to address the hypothesis being tested.**

Authors: Thanks

**statistical analysis used was not appropriate. you should use two-way 2*2 repeated measure ANOVA to assess the main effects and interaction between group and postural condition for each balance parameters. Please calculate effect sizes for each statistical analysis performed.
There are not concerns about ethical or regulatory requirements being met.**

Authors: Thanks. Thank you for your feedback on our manuscript and your suggestion to use a two-way 2x2 repeated measures ANOVA to assess the main effects and interactions between group and postural condition for each balance parameter. After careful consideration, we believe that our chosen statistical approach is more appropriate for the nature of our study and data. In our research, each participant was evaluated under two postural conditions (eyes open and eyes closed), and the results were compared between two independent groups (leprosy and control). While repeated measures ANOVA is suitable for analyzing interactions between factors within the same subjects over different conditions, our study design involves comparing two independent groups in two different states. This setup makes a non-parametric test approach, such as the Mann-Whitney test, more suitable for comparing independent groups. Additionally, using repeated measures ANOVA could introduce unnecessary complexity in the analysis and interpretation of our results, particularly in assessing interactions between factors that may not be clear or significant in this context. Given the simplicity of our experimental design—comprising two groups in two conditions—a direct comparison test like the Mann-Whitney test provides a more straightforward and interpretable analysis for addressing our specific research questions. Moreover, our data did not meet the assumptions required for repeated measures ANOVA. The Shapiro-Wilk test indicated a non-normal distribution of the data, and repeated measures ANOVA assumes that the data follow a normal distribution and that there is homogeneity of variances, conditions not met by our dataset. For these reasons, we believe our approach is statistically sound and aligned with the nature of our study. We appreciate your suggestion and have carefully considered it. However, based on the reasons outlined above, we believe it is in the best interest of the study's validity to maintain the current statistical approach.

**Results**
**Reviewer #2: Table 1 displays " muscle strength " as a demographical feature of the sample, but only for the leprosy participants. Why was this subject important enough to be on the table, since it was not described at the methodology section, nor was mentioned at the introduction? And Why was the control Group not evaluated for muscle strength as well?**

Authors: Thank you for your feedback regarding the inclusion of "muscle strength" in Table 1.

We included muscle strength data, in the previous version, as a clinical feature for the leprosy participants to document their clinical condition. However, we recognize that using this method to assess muscle strength in healthy individuals, such as those in the control group, would likely result in maximum scores of 5, as they are expected to have normal muscle strength. Since this data does not contribute meaningfully to the study's results, hypotheses, discussion, or conclusions, we have decided to remove it from the table. This change aims to avoid any confusion for readers and to keep the focus on the relevant findings of our research. Thank you for bringing this to our attention.

**The correlation between platform and sensors should be better described in the text.**

Authors: Thanks for your contribution. We’ve included it

**Reviewer #3: Please consider comment about statistical analysis described above and based on revise the Results section.**

Authors: Thanks for your contribution. It was previously answered.

**the figures (Tables, Images) are sufficient quality for clarity**

Authors: Thanks.

**Conclusions**

**Reviewer #2: commentaries already given on " Methods" section**

Authors: Thanks for your contribution. It was previously answered.

**Reviewer #3: the conclusions are supported by the data presented.**

**the limitations of analysis are partly described.**

**the authors discusses how these data can be helpful to advance our understanding of the topic under study.**

**public health relevance is addressed**

Authors: Thanks for your contribution.

**Summary and General Comments**

**Reviewer #2: The reference # 11 dos not cite the disability grade as described in this manuscript Please review the need to cite this. Reference**

Authors: Thank you for your feedback regarding the use of reference #11 in our manuscript. We believe that reference #11 is important to include as it supports the rationale for evaluating balance even in patients without apparent disability. This reference discusses the concept of "silent neuropathy" or "quiet nerve paralysis," highlighting that significant sensory and motor impairments can occur in the absence of typical clinical signs or visible disability. Including this reference helps to substantiate our approach by providing evidence that leprosy patients may have underlying neurological impairments affecting balance, even when they do not exhibit overt disability signs. Therefore, we feel that citing this reference is necessary to justify our focus on balance assessment in this population, reinforcing the importance of early detection and monitoring of neurological alterations, regardless of the visible disability grade.

**Reviewer #3: This study was aimed to evaluate the static balance of participants with multibacillary leprosy and healthy participants using a force platform and an inertial sensor, and to perform clinical and concurrent validation of the inertial sensor. This research confirmed the concurrent validity of the inertial sensor with the force platform and its clinical validation, demonstrating that this instrument can be applied in clinical settings due to its low cost and ease of use. Overall, the study is interesting, however there are some clarifications needed.**

**Comment#1
Title: Title is not appropriate. Please edit it**

Authors: Thanks for your contribution. It was previously answered.

**Comment#2
Abstract. Please state Discussion more briefly and precisely.**

Authors: Thanks for your contribution. We have corrected that.

**Comment#3
Methods. Design of this study is case-control not cross-sectional.**

Authors: Thanks for your contribution. We have corrected that.

**Comment#4
Please explain your justification for postural conditions selected to assess postural balance.**

Authors: The postural conditions of eyes open and eyes closed were selected to assess postural balance because they are widely recognized as standard and effective methods for evaluating balance control and postural stability.

**Comment#5
Please state justification for balance parameters assessed.**

Authors: The balance parameters assessed in this study were chosen to provide a comprehensive evaluation of postural stability and control, particularly in the context of leprosy-related neuropathy. These parameters are standard in balance studies and offer insights into different aspects of balance function.

**Comment#6
Please provide reference/references for this sentence “Our COP results align with the scarce existing literature that compared healthy individuals with leprosy patients, which also demonstrated greater oscillations in the group with the pathology.”**
Authors: Thanks for your contribution. We have included that.
